# Modulation of Gut Microbiota Metabolism in Obesity-Related Type 2 Diabetes Reduces Osteomyelitis Severity

**DOI:** 10.1128/spectrum.00170-22

**Published:** 2022-03-22

**Authors:** Tina I. Bui, Ann Lindley Gill, Robert A. Mooney, Steven R. Gill

**Affiliations:** a Department of Microbiology and Immunology, University of Rochester School of Medicine and Dentistry, Rochester, New York, USA; b Department of Pathology and Laboratory Medicine, University of Rochester School of Medicine and Dentistry, Rochester, New York, USA; c Center for Musculoskeletal Research, University of Rochester School of Medicine and Dentistry, Rochester, New York, USA; University of Nebraska-Lincoln

**Keywords:** obesity, type 2 diabetes, *Staphylococcus aureus*, osteomyelitis, gut microbiota, oligofructose, polyamines

## Abstract

Staphylococcus aureus is an opportunistic pathogen causing osteomyelitis through hematogenous seeding or contamination of implants and open wounds following orthopedic surgeries. The severity of S. aureus-mediated osteomyelitis is enhanced in obesity-related type 2 diabetes (obesity/T2D) due to chronic inflammation impairing both adaptive and innate immunity. Obesity-induced inflammation is linked to gut dysbiosis, with modification of the gut microbiota by high-fiber diets leading to a reduction in the symptoms and complications of obesity/T2D. However, our understanding of the mechanisms by which modifications of the gut microbiota alter host infection responses is limited. To address this gap, we monitored tibial S. aureus infections in obese/T2D mice treated with the inulin-like fructan fiber oligofructose. Treatment with oligofructose significantly decreased S. aureus colonization and lowered proinflammatory signaling postinfection in obese/T2D mice, as observed by decreased circulating inflammatory cytokines (tumor necrosis factor-α [TNF-α]) and chemokines (interferon-γ-induced protein 10 kDa [IP-10], keratinocyte-derived chemokine [KC], monokine induced by interferon-γ [MIG], monocyte chemoattractant protein-1 [MCP-1], and regulated upon activation, normal T cell expressed and presumably secreted [RANTES]), indicating partial reduction in inflammation. Oligofructose markedly shifted diversity in the gut microbiota of obese/T2D mice, with notable increases in the anti-inflammatory bacterium Bifidobacterium pseudolongum. Analysis of the cecum and plasma metabolome suggested that polyamine production was increased, specifically spermine and spermidine. Oral administration of these polyamines to obese/T2D mice resulted in reduced infection severity similar to oligofructose supplementation, suggesting that polyamines can mediate the beneficial effects of fiber on osteomyelitis severity. These results demonstrate the contribution of gut microbiota metabolites to the control of bacterial infections distal to the gut and polyamines as an adjunct therapeutic for osteomyelitis in obesity/T2D.

**IMPORTANCE** Individuals with obesity-related type 2 diabetes (obesity/T2D) are at a five times increased risk for invasive Staphylococcus aureus osteomyelitis (bone infection) following orthopedic surgeries. With increasing antibiotic resistance and limited discoveries of novel antibiotics, it is imperative that we explore other avenues for therapeutics. In this study, we demonstrated that the dietary fiber oligofructose markedly reduced osteomyelitis severity and hyperinflammation following acute prosthetic joint infections in obese/T2D mice. Reduced infection severity was associated with changes in gut microbiota composition and metabolism, as indicated by increased production of natural polyamines in the gut and circulating plasma. This work identifies a novel role for the gut microbiome in mediating control of bacterial infections and polyamines as beneficial metabolites involved in improving the obesity/T2D host response to osteomyelitis. Understanding the impact of polyamines on host immunity and mechanisms behind decreasing susceptibility to severe implant-associated osteomyelitis is crucial to improving treatment strategies for this patient population.

## INTRODUCTION

Individuals with obesity-related type 2 diabetes (obesity/T2D) are at increased risk for invasive Staphylococcus aureus infections as a result of obesity-induced chronic inflammation, immune cell dysfunction, and impaired immune defenses (1–4). Osteomyelitis in this patient population is severe, with infections often becoming chronic and requiring revision surgeries for (i) debridement and implant retention, (ii) debridement with implant replacement, or (iii) debridement with implant removal, resulting in greater morbidity and mortality (5). Obese/T2D individuals undergo total joint arthroplasties at a higher rate than lean, nondiabetic individuals, with data from 2011 suggesting that more than 80% of patients undergoing joint replacements are obese (6, 7). In general, periprosthetic joint infections following primary joint replacement surgeries are low (1 to 3%), but obese/T2D patients have a 5-fold increased risk for occurrence of persistent infections in up to 30% of cases (6, 8–10). Furthermore, the majority of implant-associated infections are caused by methicillin-resistant Staphylococcus aureus (MRSA) (11–14). In an era of decreased antibiotic efficacy due to bacterial resistance, exploring other avenues that target and enhance host response is urgently needed.

Impaired immunity in obese/T2D increases susceptibility to invasive infections (15–22). Obese/T2D hosts are immunocompromised, whereby chronic inflammation induced by adipokines, such as tumor necrosis factor-α (TNF-α), leads to an accumulation of senescent cells (4, 23). Neutrophils isolated from diabetic rodents and patients are impaired in all steps of their response, including adherence, chemotaxis, phagocytosis, and killing (15, 17, 21, 22). Obesity/T2D also modifies T cell function and humoral response to S. aureus osteomyelitis (24, 25). Elucidation of functions in obesity/T2D that contribute to inflammation and exacerbation of S. aureus osteomyelitis will improve understanding of the effects on infection severity (4).

Dysbiosis of the gut microbiome in obesity/T2D is a significant contributor to chronic inflammation and insulin resistance (26, 27). Gut dysbiosis is a shift in the gut microbiota composition and metabolism away from a baseline state that is linked to disease (28–30). Gut dysbiosis is influenced to a greater extent by obesity than by diabetes in obese/T2D patients, with significant changes in host metabolism (31). In obesity-related dysbiosis, overall diversity of the gut microbial community is reduced along with compositional changes, leading to higher energy harvest from diet and subsequent total body fat accumulation (26). Compelling evidence suggests that disruption of the gut microbial community by a high-fat diet leads to increased inflammation in obesity/T2D (32–34). This shift in the gut microbiota of obese/T2D rodents and individuals is linked to increased circulating lipopolysaccharide (LPS), a bacterial cell wall component recognized by toll-like receptor 4 (TLR4) (33–35). Persistent activation of TLR4 presented on innate immune cells triggers the release of proinflammatory cytokines, which promote chronic inflammation and other complications of obesity (36).

Obesity-induced gut dysbiosis is reversed by changes in the host diet, a primary driver of gut microbiota composition and metabolism (32). One way to modulate the obese/T2D gut microbiota is through indigestible dietary fiber composed of fermentable carbohydrates (37). Mammalian hosts do not possess enzymes to hydrolyze these carbohydrates and can only process and absorb nutrients after they are catabolized by gut microbes (38). Prior studies exploring the effects of an inulin-like fructan, oligofructose (OF), have demonstrated its importance in improving systemic complications of obesity/T2D, including weight loss, glycemic control, insulin resistance, and inflammation (39–43). Fermentation of oligofructose by gut microbes in the large colon produces numerous metabolic products, including short-chain fatty acids (SCFAs), which affect a broad range of cellular and immune functions (44–46). In our study, obese/T2D mice with tibial S. aureus infections were treated with oligofructose to reverse gut dysbiosis and reduce inflammation, which exacerbates infections. We hypothesized that alteration of the obese/T2D gut microbiota would lead to improvements in osteomyelitis severity through changes in bacterial metabolite production. Our results demonstrate that oligofructose supplementation resulted in reduced bone infection severity in obese/T2D mice, which was associated with alterations in the gut microbiota and downregulation of inflammation 14 days postinfection. Importantly, we identified a novel role for polyamines (PA) as beneficial metabolites that directly reduced bone infection severity similar to oligofructose.

## RESULTS

### Oligofructose decreased S. aureus implant-associated infection severity in obese/T2D mice.

**FIG 1:**
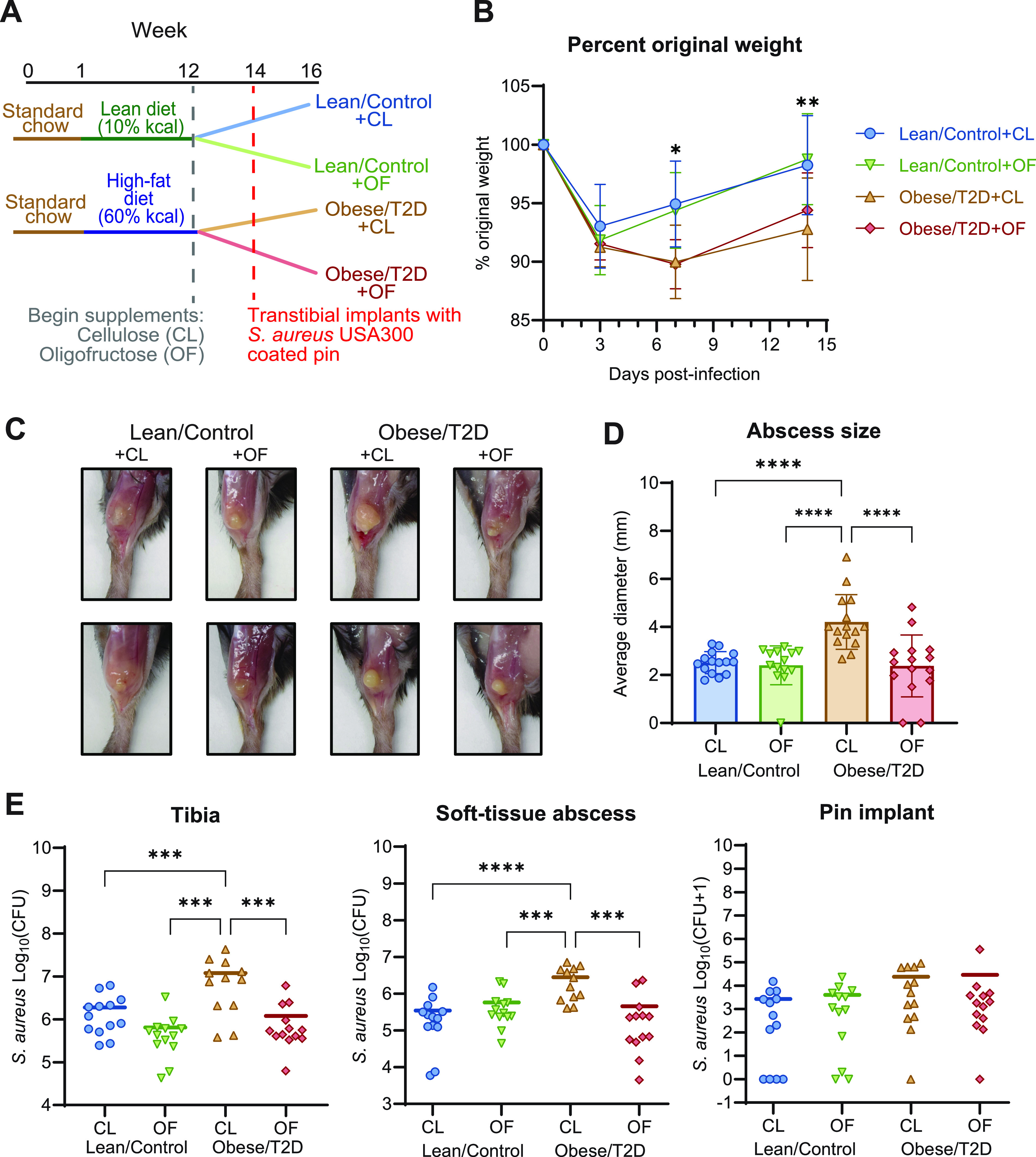
Oligofructose decreased S. aureus burden during implant-associated osteomyelitis in obese/T2D mice. (A) Schematic of the 16-week experimental timeline; CL, cellulose (control fiber); OF, oligofructose. Lean/control and obese/T2D refer to the disease state, and CL (cellulose, control fiber) or OF (oligofructose, experimental fiber) refer to the dietary supplement. For example, lean/control treated with cellulose is denoted as lean/control + CL, while control obese/T2D treated with cellulose is obese/T2D + CL. (B) Percent original weight of mice during the course of infection. Significance between lean/control + CL and obese/T2D + CL (*, *P* < 0.05; **, *P* < 0.01) was determined by two-way ANOVA with a Tukey’s *post hoc* test. There were no differences between obese/T2D + CL and obese/T2D + OF mice. (C) Two representative images of soft tissue abscesses from each treatment group 14 days postinfection. (D) Quantification of abscess size using digital caliper. (E) Infected soft tissue, tibias, and pins were collected at day 14 postinfection for enumeration of S. aureus. Bar graphs represent mean ± standard deviation (SD); *n* = 13. Significance was identified by one-way ANOVA and Tukey’s *post hoc* multiple-comparison test; **, *P* < 0.01; ***, *P* < 0.001; ****, *P* < 0.001.

Oligofructose (OF) is a soluble fiber that has been reported to alleviate some of the secondary complications of obesity and type 2 diabetes in humans and rodents (39–43). To determine if oligofructose has an effect on implant-associated osteomyelitis severity in obese/T2D mice, we used a diet-induced murine model of obesity/T2D and supplemented lean and high-fat diets with oligofructose for 2 weeks prior to and during infection (Fig. 1A). The insoluble fiber cellulose (CL) was used as a control supplement that is poorly fermented by gut microbes and resistant to mammalian metabolism (38). Results from a glucose tolerance test (GTT) performed prior to tibial transplant at week 14 demonstrated that obese/T2D + CL control mice exhibit increased glucose tolerance with a modest improvement as seen in obese/T2D + OF mice (Fig. S1 in the supplemental material). After initiation of a tibial implant-associated S. aureus USA300 infection, infections were monitored for 14 days, a time frame within which acute osteomyelitis can develop. In agreement with previous research suggesting that obese/T2D mice are at increased risk for more severe invasive osteomyelitis than healthy counterparts, the obese/T2D + CL control mice exhibited more severe infections than lean/control mice regardless of supplement, with 5% greater weight loss (Fig. 1B) and increased abscess formation and bacterial burden (Fig. 1C to E) (47, 48). Oligofructose reduced soft tissue abscess size by ∼50% (Fig. 1C and D) and the associated bacterial burden in the abscess and tibia following S. aureus implant-associated infections by ∼1 log fold compared to the obese/T2D + CL control mice (Fig. 1E). The reduction in both measures of infection with oligofructose was only observed in obese/T2D mice and not in lean/control mice. These results demonstrated that oligofructose was specifically effective in reducing infection severity within the obese/T2D subject group and therefore has therapeutic potential.

### Oligofructose reduced S. aureus community colonization in the bone.

**FIG 2:**
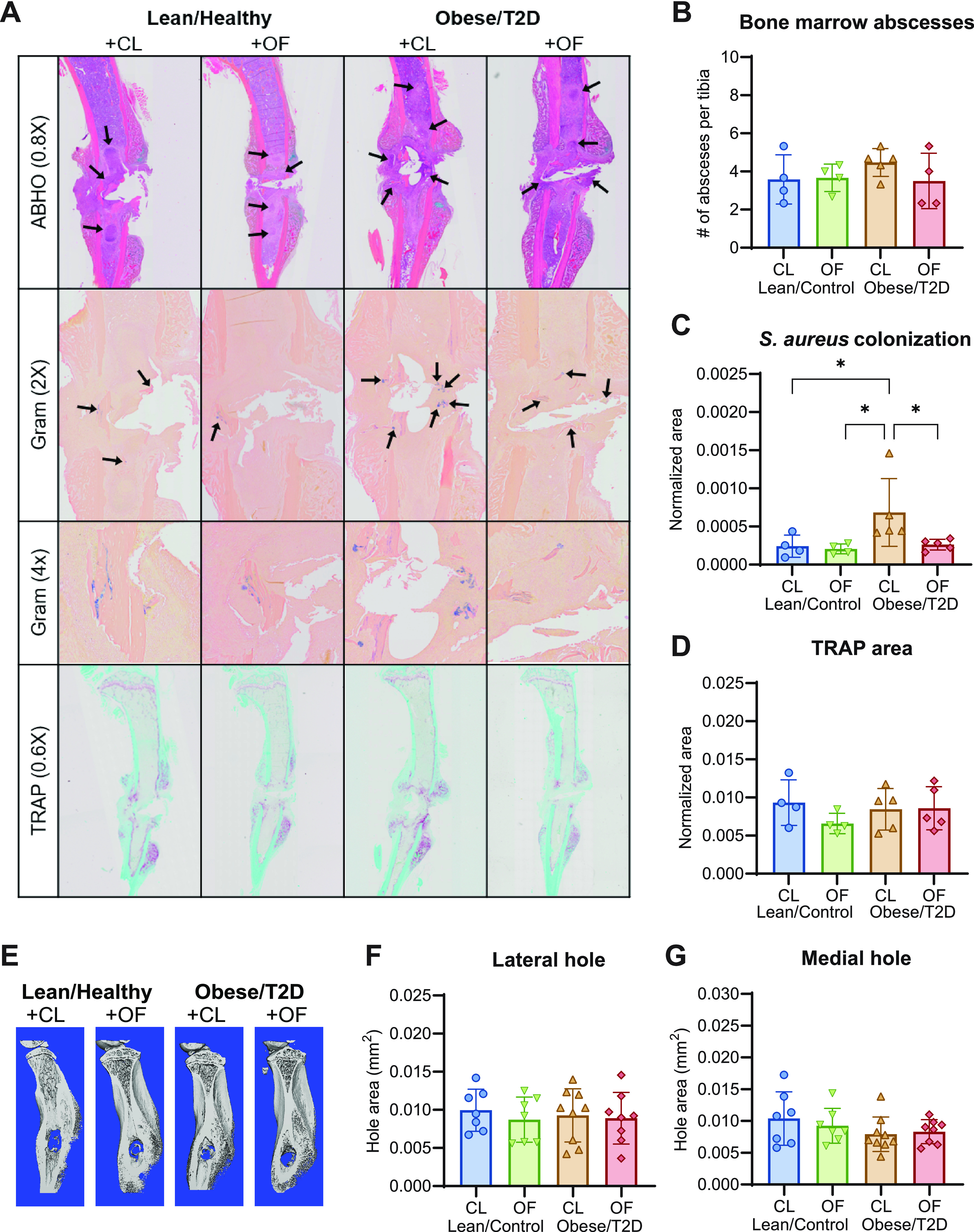
Oligofructose decreased S. aureus colonization in the bone marrow. (A) Histologic sections of infected tibias at 14 days postinfection were stained with Alcian blue hematoxylin orange G (ABHO) for visualization of abscess formation, Brown-Brenn Gram stains for S. aureus colonization, and tartrate-resistant acid phosphatase (TRAP) for osteoclast detection. (B) Quantification of bone marrow abscesses by counting across three sections per mouse. (C) Total S. aureus community colonization area in the bone marrow was quantified by using an analysis protocol package (APP) in Visiopharm, which identified positive Gram-stained area and normalized to total tibial area. At least three sections were measured per mouse. (D) TRAP area was also quantified by an APP in Visiopharm and normalized to total tibial area. At least three sections were measured per mouse. (E) Representative images of μCT analyses on infected tibias harvested 14 days postinfection. (F and G) Quantitation of lateral hold area (F) and medial hole area (G). Bar graphs represent mean ± SD; histology, *n* = 7 to 8; μCT, *n* = 5. Significance was identified by one-way ANOVA and Tukey’s *post hoc* multiple-comparison test; *, *P* < 0.05; CL, cellulose (control fiber); OF, oligofructose.

Spatial colonization of S. aureus in the bone marrow and bone osteolysis was examined as an additional outcome of osteomyelitis severity. Infected tibias were sectioned and stained with (i) Brown-Brenn Gram stain to visualize S. aureus communities in crystal violet, (ii) Alcian blue hematoxylin-orange G (ABHO) to visualize mature calcified bone in orange to red and soft tissues in pink to red for abscesses, and (iii) tartrate-resistant alkaline phosphatase (TRAP) for osteoclast activation in pink to indicate areas of osteolysis (Fig. 2A). Histological analysis showed that while the numbers of abscesses were similar across treatment groups (Fig. 2B), the total areas colonized by S. aureus in an abscess community or in a biofilm associated with necrotic bone differed. Consistent with bacterial CFU quantification of the bone (Fig. 1E), we observed ∼60% increased amounts of S. aureus communities in the bone marrow of obese/T2D + CL control mice compared to obese/T2D + OF and lean/control mice when normalizing positive Gram stain areas to whole tibia area (Fig. 2C). Analysis of osteoclast activation by TRAP staining showed no differences in osteolysis across groups (Fig. 2D). This corresponded with microcomputed tomography (μCT) imaging of the implant site (Fig. 2E) and quantification of the infected hole size for osteolysis (Fig. 2F and G). Together, these data suggest that obesity/T2D may not impact osteolysis during acute osteomyelitis (14 days postinfection). Importantly, oligofructose modestly reduced S. aureus community colonization in the infected bone.

### Oligofructose downregulates inflammatory signals in obese/T2D mice following infection.

**FIG 3:**
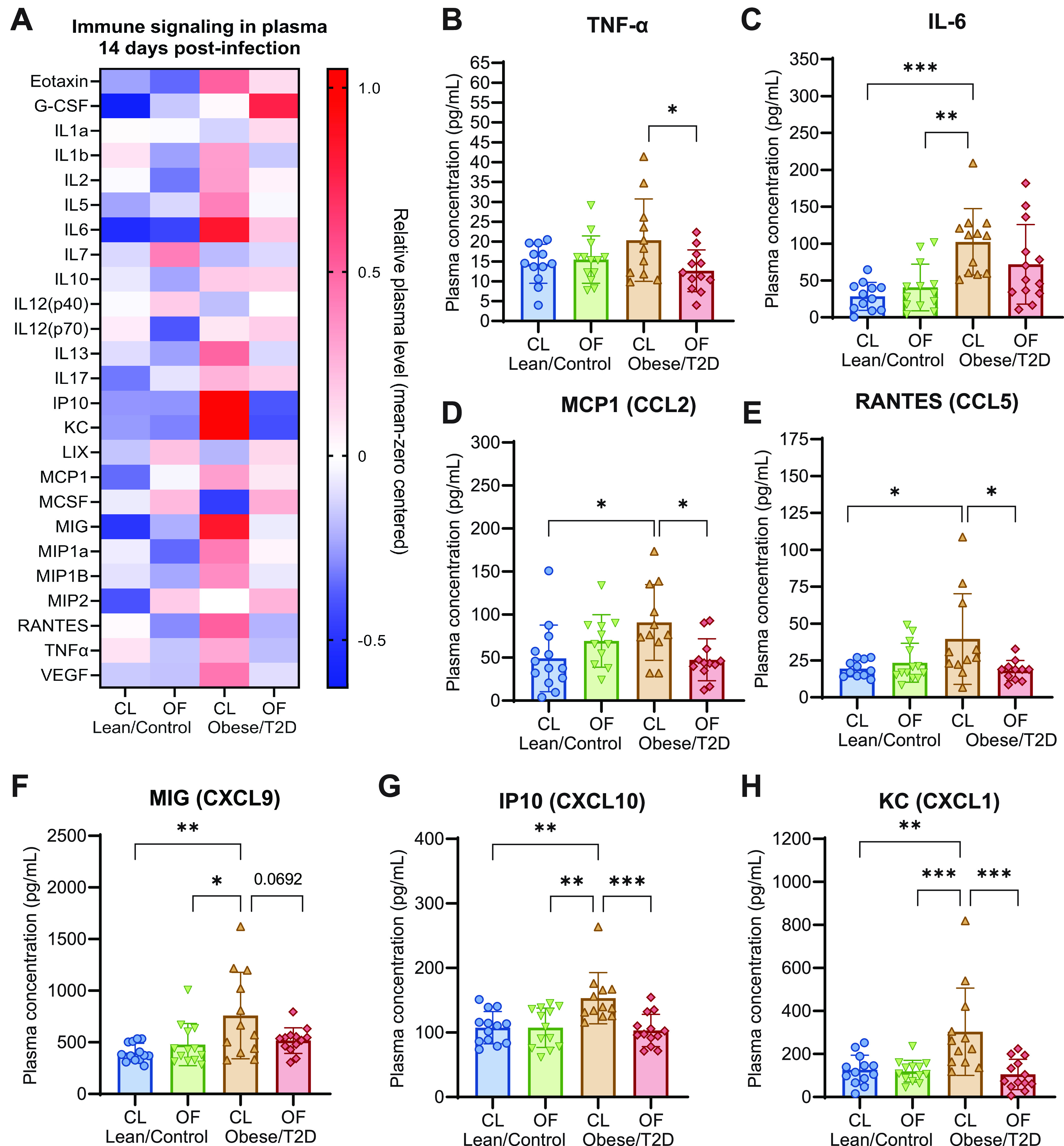
Oligofructose downregulated inflammatory signals in obese/T2D mice following infection. (A) Heat map of mean zero-centered concentrations of different cytokines/chemokines analyzed from plasma isolated 14 days postinfection. (B to H) Quantitation of significantly altered cytokines and chemokines. Bar graphs represent mean ± SD; *n* = 13. Significance was identified by one-way ANOVA and Tukey’s *post hoc* multiple-comparison test; *, *P* < 0.05; **, *P* < 0.01; ***, *P* < 0.001; CL, cellulose (control fiber); OF, oligofructose.

It is well established that the host mounts a temporary inflammatory response during acute osteomyelitis as opposed to persistent low-grade inflammation in chronic infections (49, 50). However, chronic inflammation combined with infection in obesity/T2D can lead to hyperinflammatory responses manifesting in the form of increased immune cell recruitment and cytokine storm, which then exacerbate infections (20, 51–53). To examine the effects of oligofructose on immune signaling associated with reduced infection severity, we next determined the levels of cytokines at 14 days postinfection. The obese/T2D + CL control mice displayed a hyperinflammatory immune response with overall increases in cytokines and chemokines postinfection that were not present in other treatment groups (Fig. 3A). We observed a sustained production of TNF-α and interleukin-6 (IL-6) cytokines at 14 days postinfection in the obese/T2D + CL control mice that was absent in both lean/control groups and obese/T2D + OF mice (Fig. 3B and C). Consistent with this reduction in inflammation, obese/T2D + OF mice showed a markedly decreased expression of several chemokines compared to obese/T2D control mice, including keratinocyte-derived chemokine (KC; CXCL1), monocyte chemoattractant protein 1 (MCP-1; CCL2), monokine induced by interferon-γ (IFN-γ) (MIG; CXCL9), IFN-γ-induced protein 10 kDa (IP-10; CXCL10), and regulated upon activation, normal T cell expressed and presumably secreted (RANTES; CCL5) (Fig. 3D to H). MIG and IP-10 are recruitment signals produced when induced by IFN-γ, both targeting activated T cells via chemokine receptor 3 (54). KC and MCP-1 primarily target innate immune cells, including neutrophils and monocytes, respectively (55–57). From these results, we conclude that oligofructose reversed hyperinflammation that is associated with severe osteomyelitis and higher morbidity in control obese/T2D mice at 14 days postinfection.

### Oligofructose shifted obese/T2D gut microbiota away from obesity-related dysbiosis.

**FIG 4:**
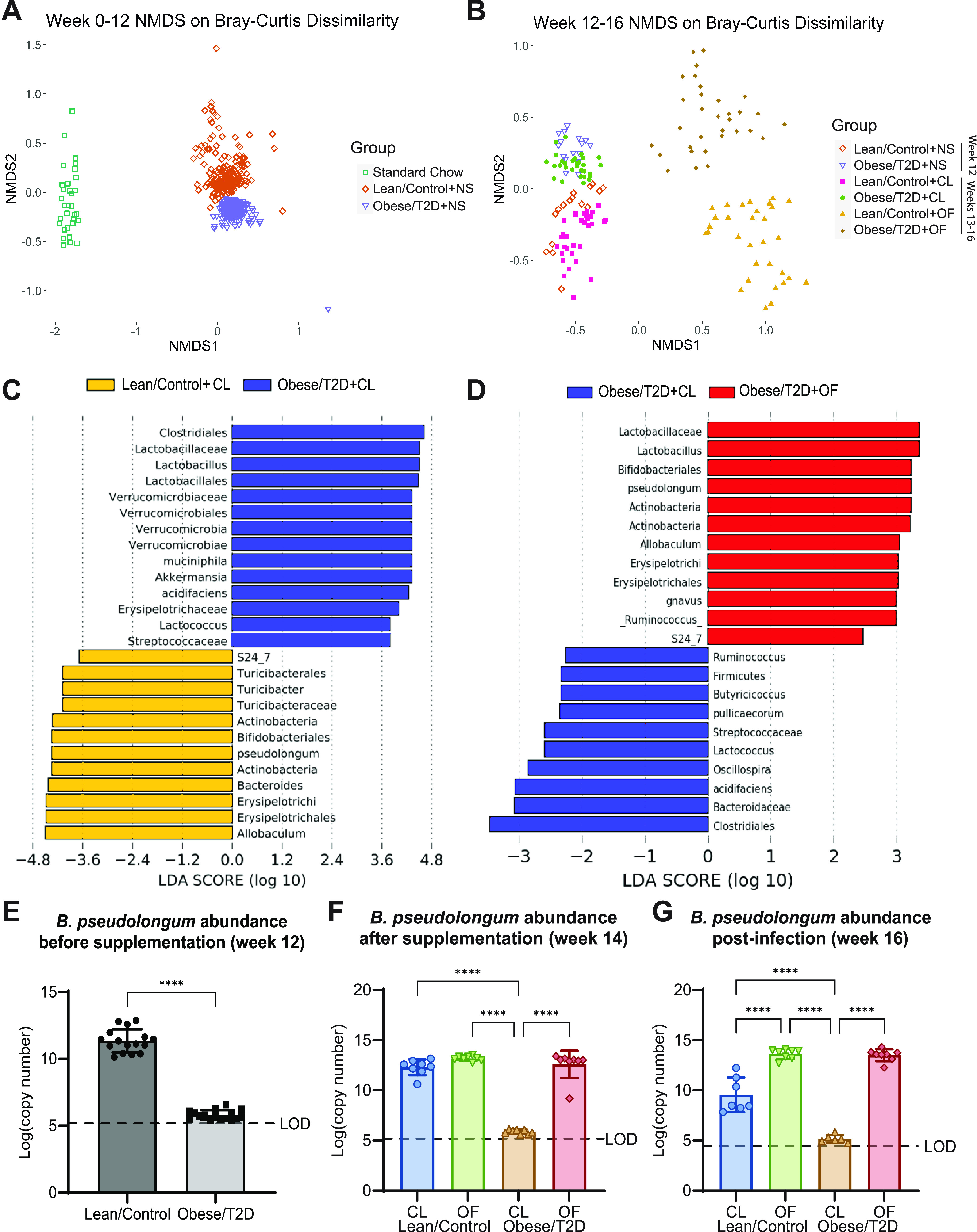
Oligofructose shifted gut microbiota away from diseased state by inducing the expansion of *Bifidobacteria pseudolongum*. (A) Nonmetric multidimensional scaling (NMDS) plot of Bray-Curtis dissimilarity metric during weeks 0 to 12; NS, no supplement. (B) NMDS plot of Bray-Curtis dissimilarity during weeks 12 to 16. Week 12 represents mice prior to supplementation; NS, no supplement. Weeks 13 to 14 represent mice supplemented with CL (cellulose, control fiber) or OF (oligofructose) before infection and during infection weeks 15 to 16. (C and D) Pairwise analysis using LEfSe was used to identify OTUs discriminating between two indicated groups. Linear discriminant analysis (LDA) scores were calculated based on a subset of vectors consisting of Kruskal-Wallis tests analyzing all features and pairwise Wilcoxon test checks between groups. (E to G) Absolute abundance of B. pseudolongum identified by qPCR for weeks 12, 14, and 16. Bar graphs represent mean ± SD; LOD, limit of detection; *n* = 7 to 8. Significance was identified by one-way ANOVA and Tukey’s *post hoc* multiple-comparison test; ****, *P* < 0.0001.

We performed 16S rRNA amplicon sequencing of mouse fecal samples collected weekly from weeks 0 to 16 to identify taxonomic shifts in community composition associated with reduced infection severity in response to oligofructose. All mice on standard chow at week 0 had similar microbiota composition prior to the dietary regimen (Fig. 4A). In agreement with prior studies, obese/T2D mice on the high-fat diet with no supplement (obese/T2D + NS) exhibited gut dysbiosis, with a shift of the microbiota to a taxonomic composition distinct from lean/control + NS mice throughout weeks 1 to 12. Lean/control + CL and obese/T2D + CL mice during weeks 13 to 16 had gut microbiota compositions similar to those observed prior to supplementation at week 12 (Fig. 4B). However, following supplementation with oligofructose through weeks 13 to 16, the obese/T2D + OF microbiota was distinct from all other groups (Fig. 4B). These results collectively demonstrate that oligofructose markedly altered the obese/T2D gut microbiota, while cellulose had little effect and served as a negative control for fiber supplementation.

To identify operational taxonomic units (OTUs) discriminative of each group, we performed linear discriminant analysis effect size (LEfSe) on the gut microbiota at 14 days postinfection (week 16). Several taxa were differentially abundant between lean/control + CL mice and obese/T2D + CL control mice (Fig. 4C). The gut microbiota of obese/T2D + CL control mice was more closely associated with Akkermansia muciniphila and *Lactobacillus* spp. (Fig. 4C). Multiple *Lactobacillus* species have been associated with weight gain and a fiber-deprived diet (58–60). In contrast, lean/control + CL mice were associated with Bifidobacterium pseudolongum, an anti-inflammatory bacterium that produces immunomodulatory short-chain fatty acids (SCFAs) from fiber fermentation, and an unknown species of the S24-7 family and *Allobaculum* genus (Fig. 4C) (61, 62). Oligofructose treatment in obese/T2D + OF mice also led to increased abundance of B. pseudolongum and unknown taxa from the S24-7 family and *Allobaculum* genus (Fig. 4D and Fig. S2). Prior to supplementation at week 12, *Bifidobacteria* was present in lean/control mice at an average of 8% relative abundance compared to 0.01% in obese/T2D mice (Fig. S2A). Oligofructose supplementation at week 14 rapidly increased *Bifidobacteria* abundance to 34% in lean/control + OF mice and 18% in obese/T2D + OF mice (Fig. S2B). This expansion in *Bifidobacteria* continued while mice were given oligofructose 14 days after infection at week 16, where *Bifidobacteria* abundance was found to be at 44% and 39% in lean/control + OF and obese/T2D + OF mice, respectively (Fig. S2C). We next quantified absolute abundance of B. pseudolongum for weeks 12, 14, and 16 by quantitative PCR (qPCR; Fig. 4E to G). Without any dietary fiber supplement at week 12, obese/T2D mice had 6-log fold less B. pseudolongum than lean/control mice with values near the detection limit (Fig. 4E). After oligofructose supplementation but prior to infection (week 14), the copy number of B. pseudolongum in obese/T2D + OF increased to levels similar to those observed in lean/control mice (Fig. 4F). Following 14 days of infection, B. pseudolongum abundance remained similar to week 14. Overall, these results illustrate that oligofructose induced large, distinct shifts in the gut microbiota of obese/T2D mice away from obesity-induced dysbiosis and more similar to that of lean/control mice. The considerable changes in the abundance of B. pseudolongum and potential contribution to immunomodulation suggested a key role for this bacterium in the microbiota response of obese/T2D mice to dietary oligofructose and a reduction in infection severity associated with increased levels of SCFAs.

### Polyamine production increased in response to dietary oligofructose.

**FIG 5:**
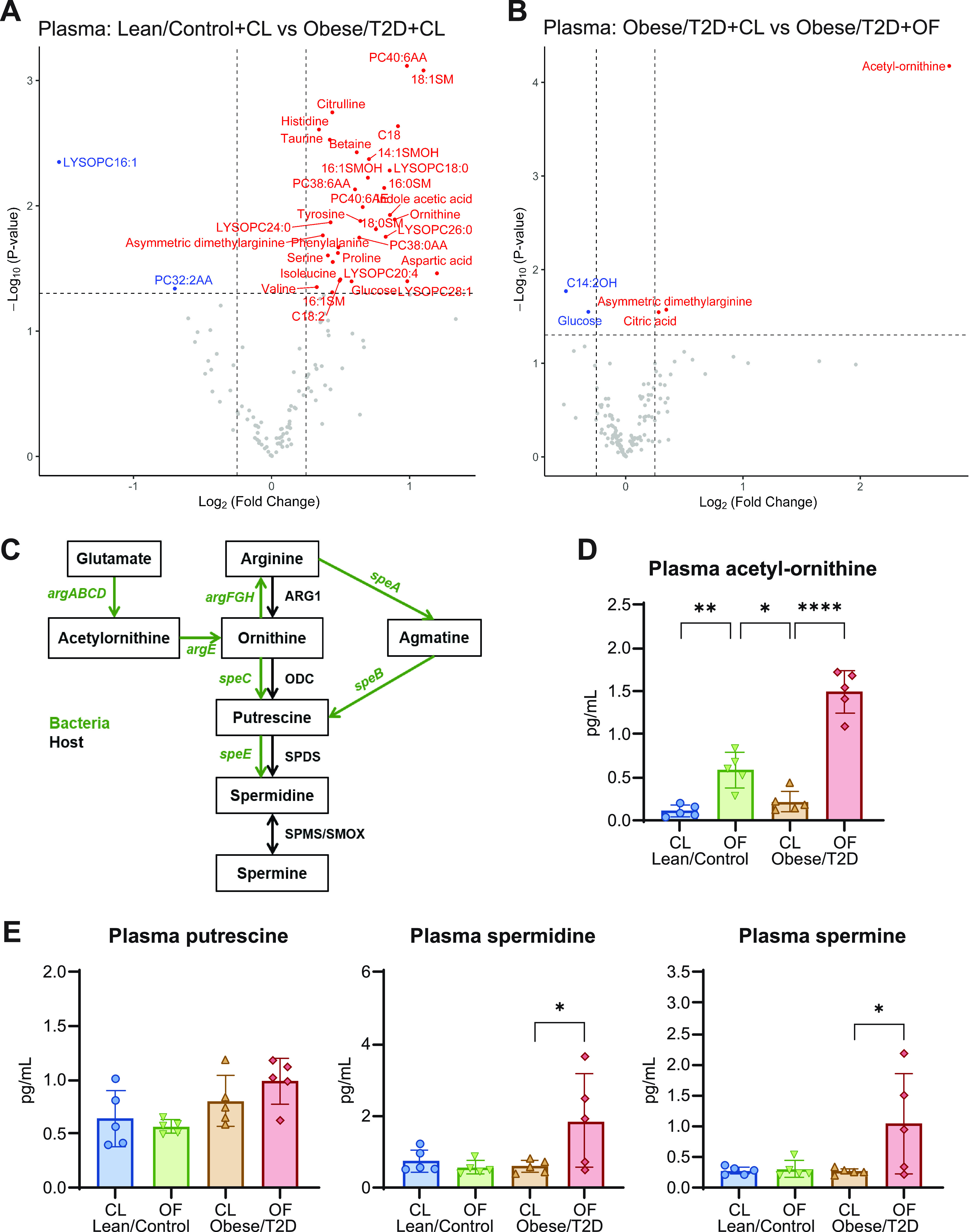
Targeted metabolomics of plasma. (A and B) Volcano plots of metabolomic profiles of plasma were compared between lean/control + CL versus obese/T2D + CL (A) and obese/T2D + CL versus obese/T2D + OF (B). Metabolites annotated in blue and red are significantly decreased and increased, respectively. (C) Mammalian and bacterial biosynthetic pathway for polyamines; ARG1, arginase 1; ODC, ornithine decarboxylase; SPDS, spermidine synthase; SPMS, spermine synthase; SMOX, spermine oxidase. (D) Absolute concentration of the polyamine precursor acetyl-ornithine. (E) Absolute concentrations of the three natural polyamines detected. Bar graphs represent mean ± SD; *n* = 5. Significance was identified by one-way ANOVA and Tukey’s *post hoc* multiple-comparison test; *, *P* < 0.05; **, *P* < 0.01; ****, *P* < 0.0001; CL, cellulose (control fiber); OF, oligofructose.

To determine which gut-microbial derived metabolite(s) were altered following oligofructose supplementation, we performed targeted metabolomics on plasma and cecal material isolated from mice after 2 weeks on supplement but before infection (Fig. 1A). Samples were harvested prior to surgery in order to identify potential biomarkers of reduced infection severity and to prevent active infections from confounding metabolite production. In the plasma, lean/control + CL and obese/T2D + CL control mice had many differentially altered metabolites, including the expected elevation of glucose in obese/T2D mice (Fig. 5A), which was in agreement with our glucose tolerance test (Fig. S1). We unexpectedly observed no differences in abundance of gut and plasma SCFAs (Fig. S3). When comparing plasma of obese/T2D + OF to plasma of obese/T2D + CL mice, a small decrease in glucose and an approximately 7-fold change in acetyl-ornithine was observed (Fig. 5B). Acetyl-ornithine, an intermediate involved in the final production of polyamines through synthesis of arginine and ornithine (Fig. 5C), was found to be significantly upregulated in both lean/control + OF and obese/T2D + OF mice (Fig. 5D). Polyamines, which are produced by both the host and gut microbes from arginine and ornithine precursors (Fig. 5C), are integral to host cellular processes, including apoptosis, proliferation, and differentiation (63). Of the three most common polyamines putrescine, spermidine, and spermine, only spermine and spermidine were found to be elevated 3- to 4-fold in obese/T2D mice fed oligofructose (Fig. 5E). Because acetyl-ornithine is microbiota derived and is used during anabolism of polyamines, increases in acetyl-ornithine following oligofructose supplementation in both lean/control and obese/T2D mice suggest that the increases in spermine and spermidine are likely derived from the microbiota.

**FIG 6:**
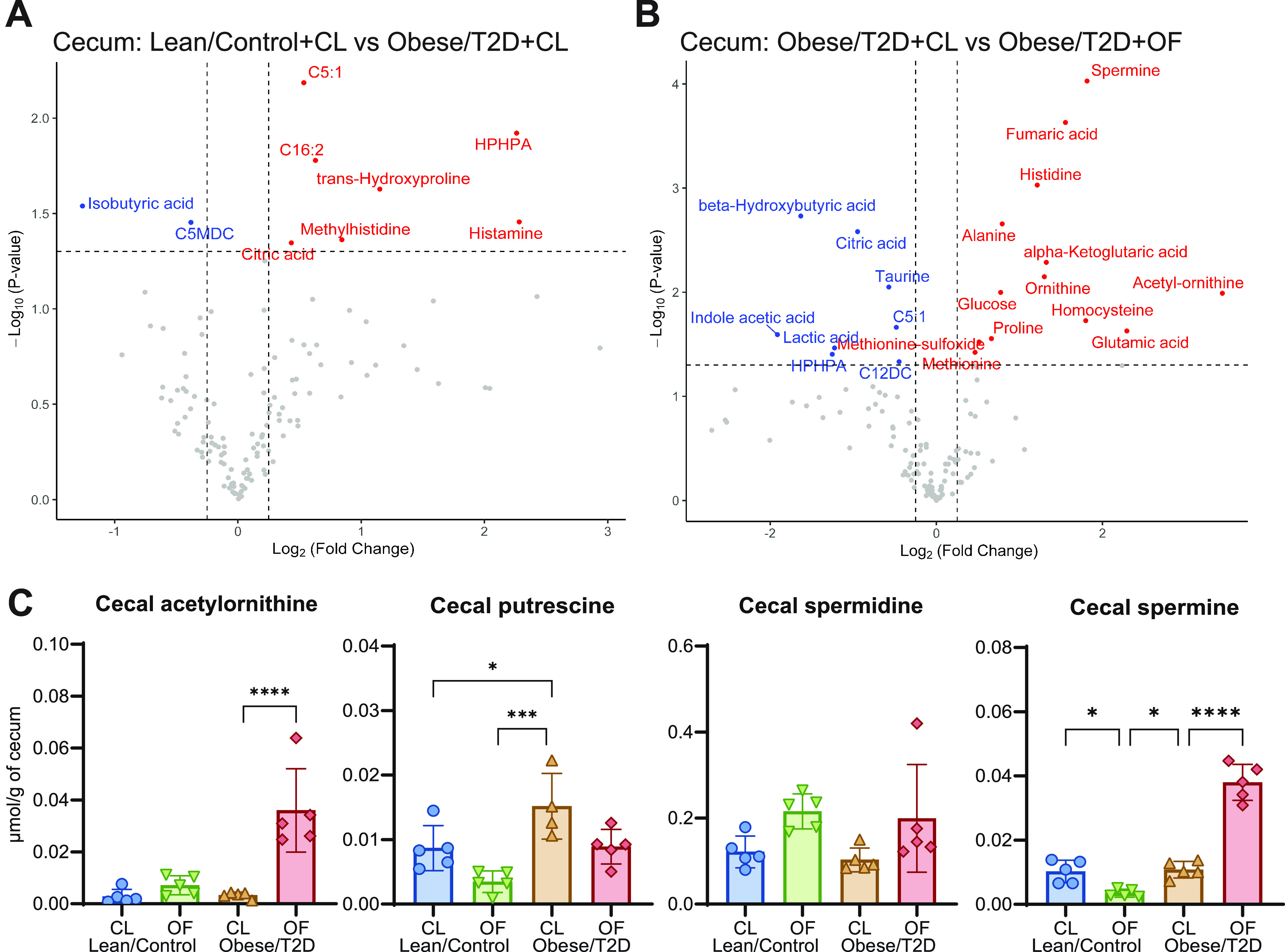
Targeted metabolomics of cecum. (A and B) Volcano plots of metabolomic profiles of cecal material were compared between lean/control + CL and obese/T2D + CL (A) and obese/T2D + CL versus obese/T2D + OF (B). Metabolite concentrations were normalized based on grams of cecal material. Metabolites annotated in blue and red are significantly decreased and increased, respectively. (C) Normalized concentrations of targeted metabolites involved in polyamine biosynthesis. Bar graphs represent mean ± SD; *n* = 5. Significance was identified by one-way ANOVA and Tukey’s *post hoc* multiple-comparison test; *, *P* < 0.05; ***, *P* < 0.001; CL, cellulose (control fiber); OF, oligofructose.

Similar trends were observed in analysis of metabolites from the cecum (Fig. 6A and B). Both spermine and acetyl-ornithine were upregulated in obese/T2D + OF mice compared to in obese/T2D + CL controls (Fig. 6B). When examining absolute concentrations of metabolites in the polyamine biosynthesis pathway, acetyl-ornithine increased by 8-fold, and spermine approximately quadrupled in obese/T2D + OF mice compared to in obese/T2D + CL control mice, while spermidine levels doubled but were not statistically significant (Fig. 6C). These results suggest that altered biosynthesis of polyamines as a result of oligofructose supplementation and shift in the microbiota may contribute to a reduction in infection severity.

### Oral administration of polyamines reduced infection severity similar to oligofructose in obese/T2D mice.

**FIG 7:**
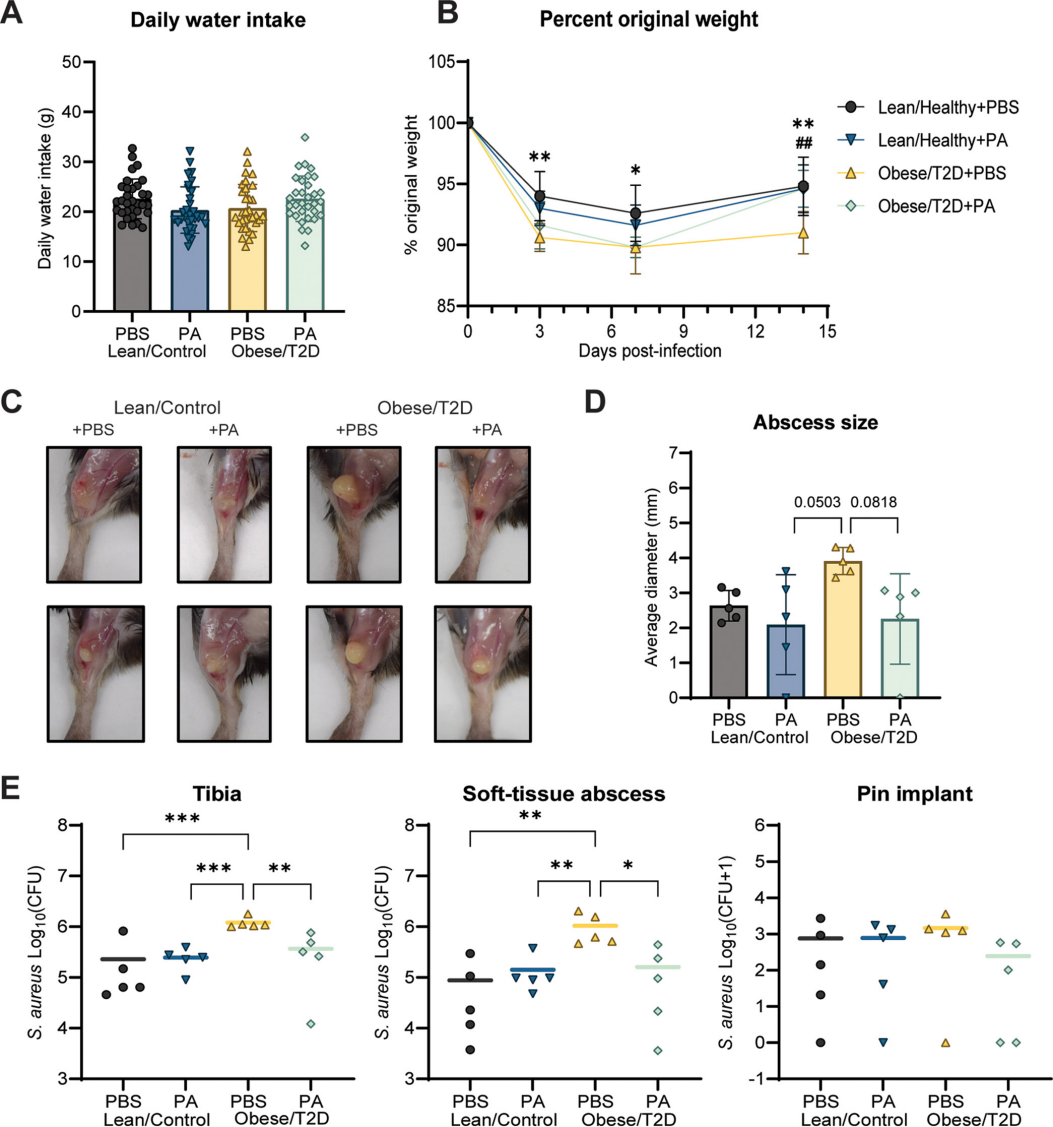
Polyamines reduced infection severity similar to the fiber oligofructose. (A) Daily water intake was determined by measuring changes in weight of drinking bottles. (B) Percent original weight of mice over 14 days of infection. Significance between lean/control + CL and obese/T2D + CL (*, *P* < 0.05; **, *P* < 0.01) and obese/T2D + CL versus obese/T2D + OF (^#^^#^, *P* < 0.01) was determined by two-way ANOVA with Tukey’s *post hoc* test. (C) Two representative images of soft tissue abscesses 14 days postinfection. (D) Quantification of soft tissue abscess size seen in C. (E) Infected soft tissue abscesses and bones were homogenized and serially diluted for bacterial quantification 14 days postinfection. Bar graphs represent mean ± SD; *n* = 5. Significance was identified by one-way ANOVA and Tukey’s *post hoc* multiple-comparison test; *, *P* < 0.05; **, *P* < 0.01; ***, *P* < 0.001; PBS, phosphate-buffered saline (control); PA, polyamines (3 mM total spermine + spermidine).

**FIG 8:**
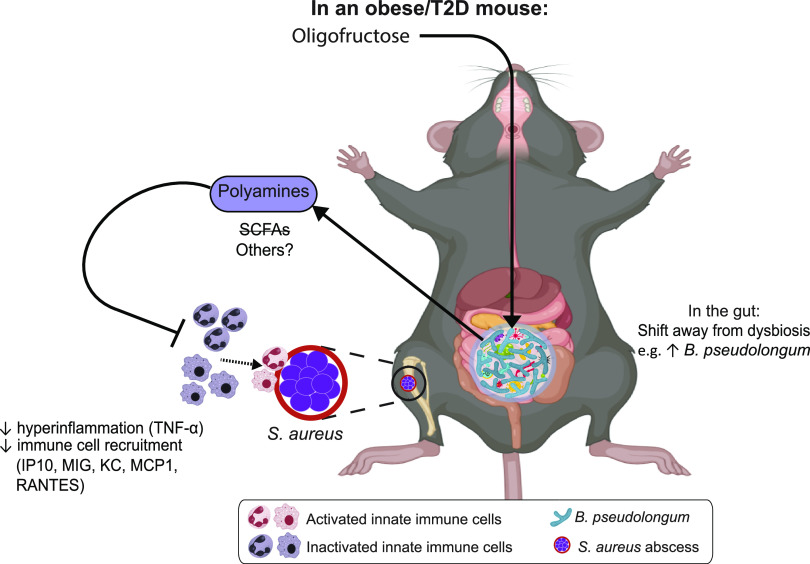
Proposed mechanism mediating the beneficial effects of oligofructose on osteomyelitis in obese/T2D mice. In an obese/T2D mouse, oligofructose supplementation led to increased *Bifidobacteria pseudolongum* in the gut and significantly shifted the gut microbiome away from a nonobese/diabetic state. This was associated with elevated levels of polyamines in the local gut environment and systemically in plasma. Oligofructose supplementation in obese/T2D mice also resulted in reduced infection severity and reduced proinflammatory signaling, suggesting a dampening of hyperinflammatory response seen in control obese/T2D mice. Direct administration of polyamines to obese/T2D mice led to similar reduction in bone infection severity to oligofructose, suggesting that polyamines are involved in modulating the obese/T2D immune responses against S. aureus*-*mediated osteomyelitis.

In order to directly determine the effects of polyamines on osteomyelitis severity, mice were supplemented with spermine and spermidine in drinking water 2 weeks prior to and continuing after infection. Water consumption in mice given polyamines (PA) and controls given phosphate-buffered saline (PBS) was similar (Fig. 7A). Weight loss in obese/T2D mice was significantly greater than that observed in lean/control mice at 3 and 7 days postinfection regardless of chemical supplement (Fig. 7B). At day 14, obese/T2D + PA mice had markedly reduced weight loss by 5% compared to obese/T2D + PBS control mice, suggesting efficacy of polyamines in overall recovery. Polyamine treatment in obese/T2D + PA mice also led to 50% smaller abscesses but had no effect in lean/control + PA mice. More importantly, polyamine supplementation decreased the elevated S. aureus bone and soft tissue burden in obese mice by approximately 1-log fold while having no effect in lean/control mice. These results demonstrate that the effects of polyamines on S. aureus osteomyelitis in obese/T2D mice were similar to those of oligofructose (Fig. 1) (64, 65). To determine whether polyamines directly inhibited the growth of S. aureus USA300 LAC::lux, we performed *in vitro* growth experiments that showed that S. aureus USA300 LAC::lux is not susceptible to polyamines (Fig. S4). Therefore, we conclude that polyamines contribute to the reduced infection severity observed in obese/T2D mice when supplemented with oligofructose through immune modulation and not direct killing of the pathogen (Fig. 8).

## DISCUSSION

A major component of host response to infections and systemic inflammation is the gut microbiota (66, 67). In obesity/T2D, gut dysbiosis contributes to chronic inflammation, which causes deficits in the immune system, thereby increasing susceptibility to and exacerbating infections (29). Thus, modulating the gut microbiota of obesity/T2D can potentially identify microbial pathways important for enhancing host immune responses against S. aureus osteomyelitis. One modifier of gut dysbiosis is dietary fiber. To explore this approach, we supplemented diet-induced obese/T2D mice with an inulin derivative, oligofructose, and examined infection outcomes, including bacterial burden, immune signaling, and metabolic profiles. To our knowledge, this is the first study to show efficacy of oligofructose in reducing infection severity during tibial implant-associated osteomyelitis, a site distal to the gut. Our results further showed that polyamines were involved in mediating improved infection severity.

We demonstrated that oligofructose decreased bacterial burden in both the infected tibia and soft tissue of obese/T2D mice compared to controls (Fig. 1E). In contrast, oligofructose did not impact soft tissue abscess size or bacterial burden in lean/control + OF mice compared to those in lean/control + CL mice, the fiber controls (Fig. 1D and E). Since lean/control mice without any supplement did not have gut dysbiosis nor the associated inflammation of obesity/T2D (Fig. 3A), the beneficial effects of oligofructose may not alter the course of infection, as lean/control mice already exhibit normal inflammatory responses. Bacterial burden on the pin implants was also not affected by oligofructose supplementation, likely due to implants acting as fomites harboring S. aureus in a biofilm state, which are physiologically different than abscesses in tissue and bone (68). Oligofructose also decreased the elevated total S. aureus community colonization in obese/T2D mice (Fig. 2C). Although prior studies have shown obese/T2D mice to have a significant impact on osteolysis, we did not observe an effect in this study (Fig. 2C and E to G), likely due to measurement at 14 versus 21 days (47, 48). Our model examined the effects of oligofructose during acute osteomyelitis, an infection that causes short-term inflammation, whereas 21 days postinfection emphasizes intermediate chronic infections with distinct characteristics, such as a fibrotic marrow and periosteal reactive bone formation (50). Nonetheless, we conclude that oligofructose had beneficial effects on bone infection severity in obese/T2D mice and warrants further investigation as a positive modifier of immunity and gut dysbiosis.

In this study, oligofructose prevented sustained inflammation in obese/T2D mice. Several proinflammatory signals that were elevated in the obese/T2D control mice were downregulated by oligofructose postinfection, including TNF-α and IL-6 and several chemotactic signals (Fig. 3). During acute infections as this model illustrates, inflammation is normally reduced so that wound healing can occur. Therefore, decreased recruitment signals for inflammatory mediators by oligofructose likely reduced tissue damage induced by inflammation, allowing for better wound healing in obese/T2D murine hosts. Additional studies to support this premise include quantification of tissue damage markers, such as lactate dehydrogenase or M2 polarization of macrophages, which are associated with inflammation resolution. Nonetheless, the elevated inflammatory signals in obese/T2D + CL control mice suggested a hyperinflammatory response to infection that may contribute to increased infection severity in this patient population. Several other studies have suggested that a hyperinflammatory response can worsen infections. Nielsen and colleagues demonstrated that obesity-related diabetes exacerbated Acinetobacter baumannii-induced sepsis, which improved following administration of the immunosuppressing agent dexamethasone (51). Genetically altered obese/T2D (*db*/*db*) mice demonstrated increased neutrophil infiltration in infections of the hind paw and heightened production of chemokines CXCL1 and CXCL2 12 h after infection (20). However, *ex vivo* experiments with peripheral neutrophils isolated from *db*/*db* mice showed diminished bacterial killing when challenged with S. aureus (20). Previous work from our group has also led to similar conclusions. We have reported that diet-induced obese/T2D mice exhibit a hyperinflammatory response with significantly more macrophages recruited to the site of infection, corresponding with increased bone destruction and bacteria survival (47). This current study provides not only additional evidence that hyperinflammatory responses seen in obese/T2D hosts are linked to more severe infections but also evidence for oligofructose moderating inflammation that would normally exacerbate acute osteomyelitis.

To determine a potential mechanism responsible for reduced inflammation and infection severity, we investigated longitudinal changes in the gut microbiota and metabolite profiles in all four groups of mice (Fig. 1A). The gut microbiota of obese/T2D mice without any supplement shifted to a dysbiotic composition distinct from lean/control mice (Fig. 4A). Long-term supplementation (3 months) of cellulose has been described to reduce gut inflammation (69). Here, 4 weeks of cellulose had minimal effects on gut microbiota composition of lean/control and obese/T2D mice, so their gut microbiota were not distinct from before cellulose supplementation (Fig. 4B). Upon supplementation with oligofructose, however, the gut microbiota of both lean/control and obese/T2D mice shifted dramatically (Fig. 4B). Discriminant analysis suggested that several taxa were altered, including B. pseudolongum and *A. muciniphila* and those from the S24-7 (also known as *Muribaculaceae*) family and *Allobaculum* genus (Fig. 4C and D). Here, we focused on B. pseudolongum as an example of the distinct shifts induced by oligofructose because (i) *A. muciniphila* was not significantly altered in obese/T2D mice given oligofructose and (ii) taxa from the S24-7 family and *Allobaculum* genus are currently uncharacterized. Both S24-7 and *Allobaculum* have been associated with fermentable fiber supplementation and improved glucose tolerance (70, 71). However, our understanding of *A. muciniphila*, *Allobaculum*, and S24-7 is lacking compared to *Bifidobacterium* spp.; thus, our ongoing efforts include isolating and exploring the beneficial effects of these microbes. Overall, oligofructose promoted significant shifts in the gut microbiome of obese/T2D mice when considering both richness and abundance, with noteworthy changes in B. pseudolongum abundance (Fig. 4D).

*Bifidobacterium* spp. are well known producers of SCFAs, immunomodulatory metabolites that are products of fiber fermentation. Similar to previous research that suggests that oligofructose induces bifidogenic effects, supplementation with oligofructose led to an expansion in B. pseudolongum (Fig. 4D) (61, 62). Unexpectedly, we did not observe elevated SCFAs in mice given oligofructose compared to cellulose (Fig. S3 in the supplemental material). However, unique to our study, we observed increased production of the natural polyamines spermine and spermidine in plasma and cecum samples of obese/T2D + OF mice (Fig. 5 and 6). Although oligofructose is known to increase SCFAs, quantification is inconsistent across rodent models, as duration of supplementation (14 days to 6 weeks) and method of isolation (feces versus cecal material, fasting versus nonfasting) varies (44, 61, 72). Short-term treatment with oligofructose (total of 4 weeks) did not affect SCFAs in obese/T2D mice in this study and in an osteoarthritis model from our group (73). Consistent with our metabolomics analysis, however, is prior documentation of increased polyamine production in rats fed oligofructose for 4 weeks (74). Polyamines are bioactive polycations derived from amino acid metabolism and are essential for cellular proliferation and differentiation. Increasing evidence suggests that polyamines regulate T cell progression, promote macrophage polarization, and reduce production of proinflammatory cytokines like TNF-α, which is consistent with our data (Fig. 4) (30, 63). Polyamines are also involved in osteogenic differentiation, with studies demonstrating exogenous polyamines disrupting osteoclast differentiation leading to attenuated bone loss (75). Therefore, polyamines may be tested to prevent bone destruction associated with chronic infections in obesity/T2D, where mice at 21 days postinfection show significantly more bone loss than lean/control mice (47). We suggest that the changes in the gut microbiota, including increased B. pseudolongum abundance, likely lead to increased systemic polyamine production for several reasons: (i) experiments with various *Bifidobacteria* species demonstrated their capability to produce spermidine *in vitro*, (ii) shotgun metagenomic sequencing of gut microbiota from obese mice fed resistant starch indicated that polyamine synthesis was associated with increases in B. pseudolongum, and (iii) treatment of mice with arginine and Bifidobacterium animalis led to increased levels of circulating and colonic polyamines, which corresponded with decreased levels of TNF-α and IL-6 (76–78). The evidence from our work demonstrated that polyamines were significantly upregulated in the gut, where microbes are abundant (Fig. 6), and the beneficial effects of polyamines on osteomyelitis in obese/T2D mice were observed when administered directly in drinking water (Fig. 7). However, S. aureus USA300 growth was not directly inhibited by spermine and spermidine (Fig. S4) due to previously characterized resistance conferred by *speG*, a gene in the arginine catabolic mobile element (64, 65). We, therefore, conclude that polyamines are immunoregulatory within the context of osteomyelitis rather than direct inhibitors of S. aureus growth based on our *in vitro* growth curve results.

In conclusion, we successfully demonstrated the therapeutic potential of the dietary fiber oligofructose on osteomyelitis in obese/T2D hosts (Fig. 8). Oligofructose reduced bacterial burden and S. aureus community colonization in the bone and ameliorated the hyperinflammatory response in obese/T2D mice, indicating that the fiber reduced infection severity. The observations corresponded with changes in the gut microbiota and overall metabolism that suggested that polyamines, and not short-chain fatty acids, were involved in improving infection response. Increased B. pseudolongum abundance and other bacteria affected by oligofructose likely contribute to heightened production of polyamines, as their increased abundance was associated with increased acetyl-ornithine, an intermediate of polyamine synthesis produced solely by bacteria. Treating obese/T2D with polyamines in drinking water provided direct evidence for the potential of spermine and spermidine in reducing infection severity. Overall, this study uncovered a novel role for oligofructose and polyamines in the context of bone infections, warranting further investigation into their role in immunoregulation and potential as adjunct therapeutics for obese/T2D hosts against invasive S. aureus-mediated osteomyelitis.

## MATERIALS AND METHODS

### Animals.

All handling of mice and associated experimental procedures were reviewed and approved by the University Committee on Animal Resources at the University of Rochester Medical Center. Male C57BL/6J mice from The Jackson Laboratory (Bar Harbor, ME) were housed five per microisolator cage in a two-way housing room on a 12-h light/dark schedule. Male mice were used, as they gain weight more consistently than female mice (79). At 6 weeks of age, mice were provided unrestricted access to either lean (10% kcal fat; OpenSource Diets, D12450J) or high-fat (60% kcal fat; OpenSource Diets, D12492) diets for 12 weeks. At week 12, mice were transitioned to supplemented lean and high-fat diets with 10% (wt/wt) cellulose (Vivapur 105, JRS Pharma LP, USA) or 10% (wt/wt) oligofructose (Orafti P95, BENEO) for 2 more weeks. All diets were supplied by Research Diets, Inc. (New Brunswick, NJ). For experiments regarding polyamines, mice were continued on either lean or high-fat diets without supplements for the entire duration of the experiment. At week 12, mice were supplemented daily with fresh sterile 3 mM (total concentration) spermine (Sigma-Aldrich, St. Louis, MO) and spermidine (Sigma-Aldrich, St. Louis, MO) or PBS in red water bottles. Bottle weights were measured daily to track drinking habits. All mice were then infected with S. aureus at week 14 using a pin model described below and monitored for an additional 2 weeks following infection. At harvest, mice were sacrificed with CO_2_ and cardiac puncture as a secondary method unless otherwise noted.

### Implant-associated infection model.

S. aureus USA300 LAC::lux, a kind gift from Tammy Kielian (University of Nebraska Medical Center), was used to infect tibias of mice. Bacteria were added to 10 mL of tryptic soy broth (TSB) and cultured overnight at 37°C with shaking at 250 rpm. Stainless steel wire (0.02 × 0.5 mm) was used to make L-shaped pin implants (4 × 1 mm), which were submerged in overnight culture for 20 min prior to implantation (∼5 × 10^5^ CFU). Mice were anesthetized with 60 mg/kg ketamine and 4 mg/kg xylazine and received preoperative extended-release buprenorphine. The right tibial area was shaved and disinfected with 70% ethanol and betadine. An ∼5-mm incision was used to uncover the tibia, and 30- and 26-gauge needles were used to drill a hole through the bone to fit the pin. Pins were inserted into the drilled holes, and the surgical site was closed with 5-0 nylon sutures.

### Glucose tolerance test (GTT).

At week 14 (2 weeks after supplementation but prior to infection), mice were fasted for 6 h during early morning, and a glucose tolerance test was performed. Mice were injected intraperitoneally with 300 mg/kg glucose in 0.9% NaCl using U-100 insulin syringes (0.5 mL 30G × 5/16; Beckton Dickinson, Franklin Lakes, NJ). Tail vein blood glucose levels were then monitored before injection and at 15, 30, 90, 120 min postinjection using a OneTouch Verio glucometer and test strips (Lifescan, Milpitas, CA).

### Soft-tissue abscess size and bacterial burden quantification.

At harvest, an incision was made at the site of infection to expose soft tissue abscesses. The shortest and longest diameters of the abscess were measured using a digital caliper (Mitutoyo, Japan), and an average of the two diameters was used as the final measurement for abscess size. For bacterial burden quantification, soft tissue abscesses and pin implants were homogenized in lysing matrix A tubes at 6.0 m/s for 40 s with an MP FastPrep-24 bead-beating system. Tibias were homogenized for an extra 40 s. Homogenized tissue and bone were then serially diluted and plated on tryptic soy agar to determine CFU.

### μCT and osteolysis analysis.

Infected tibias were fixed in 10% neutral buffered formalin at room temperature for 3 days. Samples were then rinsed in PBS and distilled water. Soft tissue and pin implants were removed prior to imaging. Three-dimensional images of infected tibias were acquired by high-resolution microcomputed tomography (vivaCT 40, Scanco Medical AG, Basserdorf, Switzerland). Tibias were scanned with the following parameters: 145-kV energy setting, 300-ms integration time, voxel size of 10.5 mm, slice increment of 10 mm, and a threshold of 210. Osteolysis was determined by quantification of the void area both from medial and lateral views.

### Histological analysis.

Following μCT imaging, fixed tibias were decalcified with 14% EDTA for 7 days. Following decalcification, samples were stored in 70% ethanol until they were processed and embedded in paraffin. Transverse sections 5 μm in size were cut and mounted on glass slides. Images were scanned with a VS120 virtual slide microscope (Olympus, Waltham, MA) and visualized using Olympia OlyVIA for abscess counting (a minimum of three technical replicates were performed per mouse). Quantification of S. aureus communities and TRAP areas were performed using analysis protocol packages (APPs) in Visiopharm v.2021.07 (Hoersholm, Denmark).

### 16S rRNA sequencing.

Samples were processed and sequenced as previously described (73). Fresh fecal samples were collected into sterile microcentrifuge tubes and frozen at −80°C until extraction. Total DNA was extracted from fecal samples using a Quick-DNA fecal/soil microbe miniprep kit (Zymo Research, Irvine, CA), and DNA concentrations were quantified with a Nanodrop. V3-V4 regions of 16S ribosomal DNA were amplified with Platinum*Taq* polymerase (Thermo Fisher Scientific;) using dual indexed primers (319F: 5′-ACTCCTACGGGAGGCAGCAG-3′; 806R: 3′-ACTCCTACGGGAGGCAGCAG-5′). Amplicons were then normalized and pooled using SequalPrep normalization plates (Thermo Fisher Scientific, Waltham, MA). Pooled library was paired-end sequenced on an Illumina MiSeq (Illumina, San Diego, CA) at the University of Rochester Genomics Research Center. Each sequencing run included: (i) positive controls consisting of a 1:5 mixture of Staphylococcus aureus, Lactococcus lactis, Porphyromonas gingivalis, Streptococcus mutans, and Escherichia coli; (ii) negative controls consisting of sterile saline; and (iii) extraction controls of saline. The background microbiota was monitored at multiple stages of sample collection and processing. All sterile saline, buffers, reagents, and plasticware used for sample collection, extraction and amplification of nucleic acid were UV irradiated to eliminate possible DNA background contamination. Elimination of potential background from the irradiated buffers, reagents, plasticware, and swabs was confirmed by 16S rRNA amplification. Data from these background negative-control samples are deposited in the SRA along with positive controls (BioProject ID PRJNA786881).

### 16S analysis.

Raw reads from the Illumina MiSeq basecalls were demultiplexed using bcl2fastq version 2.19.1 requiring exact barcode matches (80). QIIME 2021.2 was used for subsequent processing (81). 16S primers were removed, allowing 20% mismatches and requiring at least 18 bases. Cleaning, joining, and denoising were performed using DADA2 for both runs individually prior to merging. Batch A forward reads were truncated to 265 bp and reverse reads to 244 bp, error profiles were learned with a sample of 1 million reads, and a maximum expected error of two was allowed. Batch B forward reads were truncated to 246 bp and reverse reads to 216 bp, with the same error criteria as Batch A. Batches were merged prior to taxonomic classification with a pretrained naive Bayesian classifier trained with full-length 16S sequences (82, 83). Diversity analysis was done in R version 4.0.3 using phyloseq_1.32.0 (84). Taxa that could not be classified at least at the phylum level were discarded. Taxa observed in only one sample were also discarded. Samples were rarefied at a depth of 5,554 reads, which omitted a total of three samples. Faith’s phylogenetic diversity (PD) and the Shannon index were used to measure alpha diversity, and a Kruskal-Wallis test was used to assess differences. The Bray-Curtis dissimilarity index was used to measure beta diversity (85).

### Quantitative PCR.

qPCR was used to determine absolute copy numbers of the GroEL gene using primers specific for Bifidobacterium pseudolongum (B_plon-std-F: 5′-CTCAAGAACGTTGTGGC-3′; B_plon-std-R: 5′-CGGTCTTCATCACGAG-3′) (86). Briefly, 2 μL of fecal DNA from each mouse was used as the template in 25-μL total reactions of SYBR green real-time PCR master mix as instructed by the manufacturer (Qiagen, Hilden, Germany). Reactions were performed using a CFX Connect real-time PCR detection system (Bio-Rad, Hercules, CA) with the following parameters: 95°C for 3 min; 40 cycles of 95°C for 10 s and 66°C for 30 s; and a melting curve from 65°C to 95°C. A standard curve was made from B. pseudolongum ATCC 25526 genomic DNA diluted 10-fold. A trendline was fitted to a linear curve and used to calculate absolute copy numbers in fecal samples.

### Multiplexed cytokine array.

Blood samples were collected at sacrifice via cardiac puncture and stored in K_2_EDTA anticoagulant tubes (Beckton Dickinson, Franklin Lakes, NJ). To isolate plasma, blood was centrifuged at 2,000 × *g* for 10 min at 4°C. Plasma was transferred to clean microcentrifuge tubes and stored at −80°C until analysis. Plasma samples were analyzed by Eve Technologies Corporation (Calgary, AB, Canada) using a multiplex assay for levels of 31 different cytokines and inflammatory mediators. Cytokines/chemokines with more than one undetectable sample were omitted in the heat map.

### Targeted metabolomics.

A separate cohort of mice was dedicated to targeted metabolomic profiling. Mice were fed on the same feeding scheme as before but were harvested 2 weeks after supplementation without infection to prevent confounding effects of infection on metabolism. Mice were fasted for 6 h prior to intraperitoneal injection with 100 mg/kg Euthasol (phenytoin/pentobarbital) and cervical dislocation. Plasma was isolated as previously described from blood collected via cardiac punctures. Cecal material was isolated using sterile forceps and placed directly into clean, preweighed microcentrifuge tubes. Both plasma and cecal samples were snap-frozen in liquid nitrogen and stored at −80°C prior to metabolomic analysis. The Metabolomics Innovation Centre (Alberta, CA) was contracted to identify and quantify up to 150 metabolites commonly found in samples.

A targeted quantitative metabolomics approach was used to analyze the samples using a combination of direct injection (DI) mass spectrometry (MS) with a reverse-phase liquid chromatography-tandem mass spectrometry (LC-MS/MS) custom assay. This custom assay, in combination with an ABSciex 4000 QTrap (Applied Biosystems/MDS Sciex) mass spectrometer, can be used for the targeted identification and quantification of up to 150 different endogenous metabolites, including amino acids, acylcarnitines, biogenic amines and derivatives, uremic toxins, glycerophospholipids, sphingolipids, and sugars (87, 88). The method combines the derivatization and extraction of analytes and selective mass-spectrometric detection using multiple reaction monitoring (MRM) pairs. Isotope-labeled internal standards and other internal standards are used for metabolite quantification. The custom assay contains a 96 deep-well plate with a filter plate attached with sealing tape and reagents and solvents used to prepare the plate assay. Fourteen wells were used for one blank, three zero samples, seven standards, and three quality control samples. For all metabolites except organic acid, samples were thawed on ice, vortexed, and centrifuged at 13,000 × *g*. Ten microliters of each sample was loaded onto the center of the filter on the upper 96-well plate and dried in a stream of nitrogen. Subsequently, phenyl-isothiocyanate was added for derivatization. After incubation, the filter spots were dried again using an evaporator. Extraction of the metabolites was then achieved by adding 300 μL of extraction solvent. The extracts were obtained by centrifugation into the lower 96-deep-well plate, followed by a dilution step with MS running solvent. For organic acid analysis, 150 μL of ice-cold methanol and 10 μL of isotope-labeled internal standard mixture was added to 50 μL of sample for overnight protein precipitation. Then, it was centrifuged at 13,000 × *g* for 20 min. Fifty microliters of supernatant was loaded into the center of the wells of a 96-deep-well plate, followed by the addition of 3-nitrophenylhydrazine (NPH) reagent. After incubation for 2 h, butylated hydroxytoluene (BHT) stabilizer and water were added before LC-MS injection. Mass spectrometric analysis was performed on an ABSciex 4000 QTrap tandem mass spectrometry instrument (Applied Biosystems/MDS Analytical Technologies, Foster City, CA) equipped with an Agilent 1260 series ultra-high-performance liquid chromatography (UHPLC) system (Agilent Technologies, Palo Alto, CA). The samples were delivered to the mass spectrometer by an LC method followed by a DI method. Data analysis was done using Analyst 1.6.2.

### *In vitro* growth curve.

S. aureus was grown overnight in TSB at 37°C with shaking at 250 rpm. Overnight culture was washed twice in PBS and normalized to an optical density (OD) of 0.5 in TSB. The normalized bacteria solution was further diluted 1:200 with a total volume of 200 μL in TSB alone or TSB with supplemented polyamines (3 mM spermine and/or spermidine) in a flat-bottom 96-well plate. Absorbance readings at 600 nm were then monitored every 30 min using a kinetic reading with a Biotek ELx808 microplate reader (Agilent Technologies, Santa Clara, CA) set at 37°C with medium shaking. Growth curves were performed in triplicates.

### Statistical analysis.

Statistical analyses for 16S rRNA sequencing and metabolomics were performed as described above. For weight assessment, the glucose tolerance test, bacterial burden analysis, histology, μCT, individual plasma cytokine assessment, B. pseudolongum copy number quantitation, and individual metabolite analysis, a one-way analysis of variance (ANOVA) with a Tukey’s multiple comparisons *post hoc* test was performed. All statistics were analyzed using GraphPad Prism 9.3.1 except for 16S rRNA sequencing and metabolomics data, which were analyzed in RStudio 4.0.3. Differences between groups were considered significant with a *P* value of <0.05. *P* values less than 0.1 are included in figures to denote trending changes.

### Data availability.

Illumina 16S rRNA V3-V4 amplicons were deposited in the SRA under BioProject ID number PRJNA786881, including positive and negative controls on each plate.
