# Supplementary material for: Modulation of Gut Microbiota Metabolism in Obesity-Related Type 2 Diabetes Reduces Osteomyelitis Severity
Source: Microbiol Spectr. 2022 Mar 22;10(2):e00170-22. doi: 10.1128/spectrum.00170-22 (PMC9045376; doi:10.1128/spectrum.00170-22)
Supplement: SUPPLEMENTAL FILE 1 — Supplemental material. Download SPECTRUM00170-22_Supp_1_seq12.pdf, PDF file, 2.2 MB [file spectrum00170-22_supp_1_seq12.pdf]

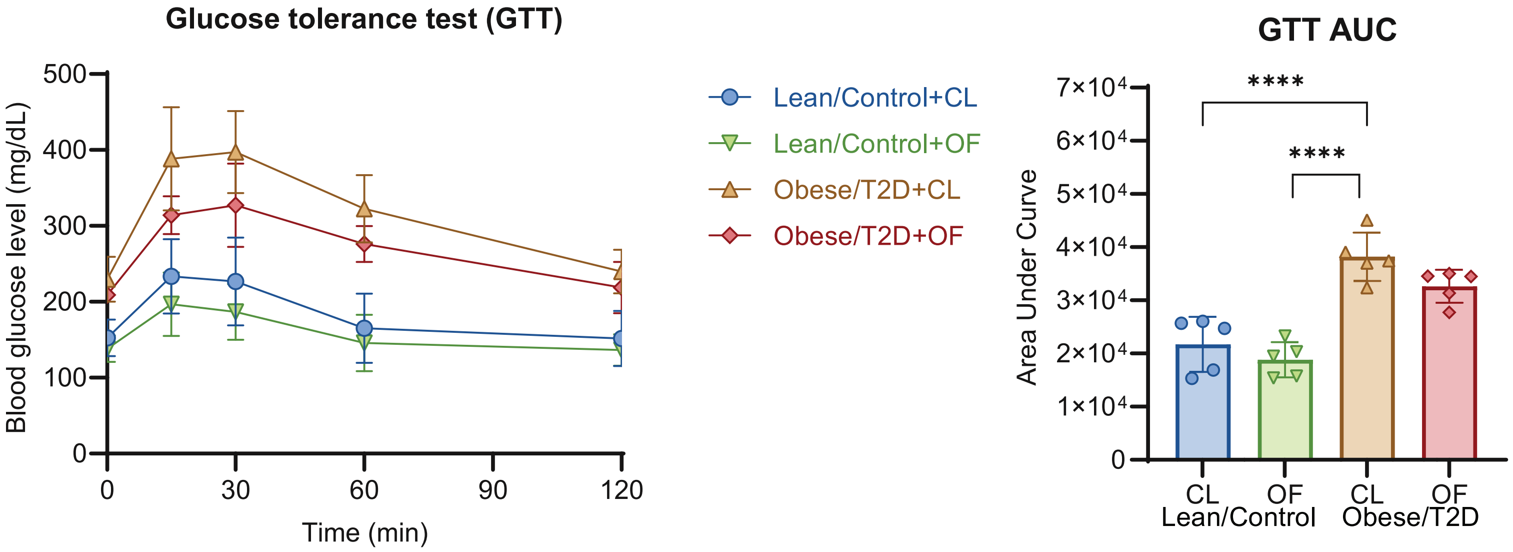

Figure S1. Diet-induced obese/T2D mice exhibited hyperglycemia compared to lean/control mice. After two weeks of supplementation with cellulose or oligofructose (week 14), mice were fasted six hours prior to glucose tolerance testing. Blood glucose levels were monitored using OneTouch Verio glucometer on blood from tail veins prior to injection with a bolus of glucose (300mg/kg). Blood glucose levels were then measured at time points = 15, 30, 60, and 120 minutes. Bar graphs represent mean ± SD. n=5. Significance was identified using one-way ANOVA and Tukey's post-hoc multiple comparisons test. **P<0.01, ***P<0.001, ****P<0.001.

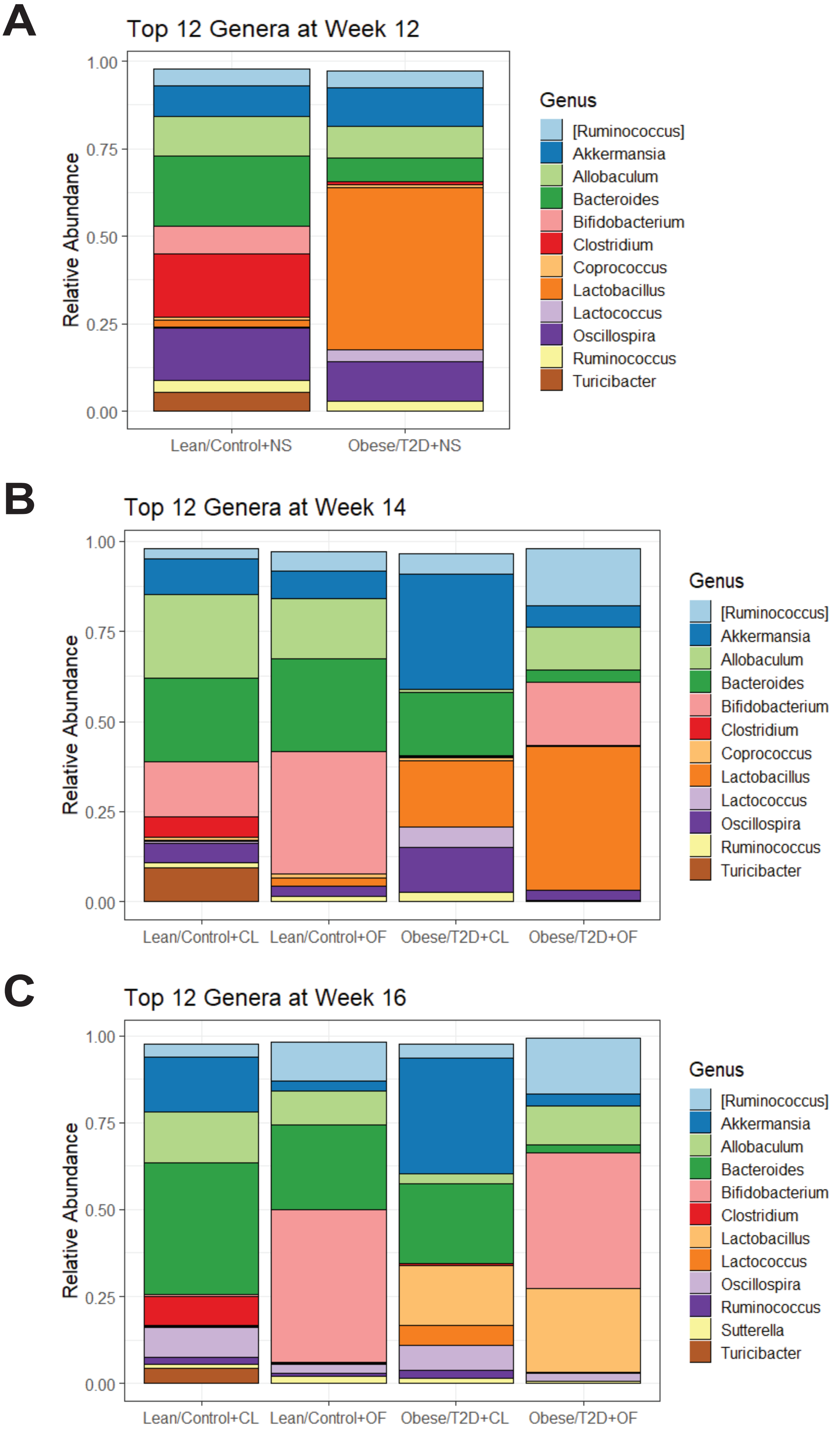

Figure S2. Top 12 genera of gut microbiota from fecal samples. A) Week 12, prior to supplementation. B) Week 14, two weeks post-supplementation with fiber but prior to infection. C) Week 16, two weeks after infection while supplemented with fiber. NS=No supplement; CL=Cellulose (control fiber); OF=Oligofructose.

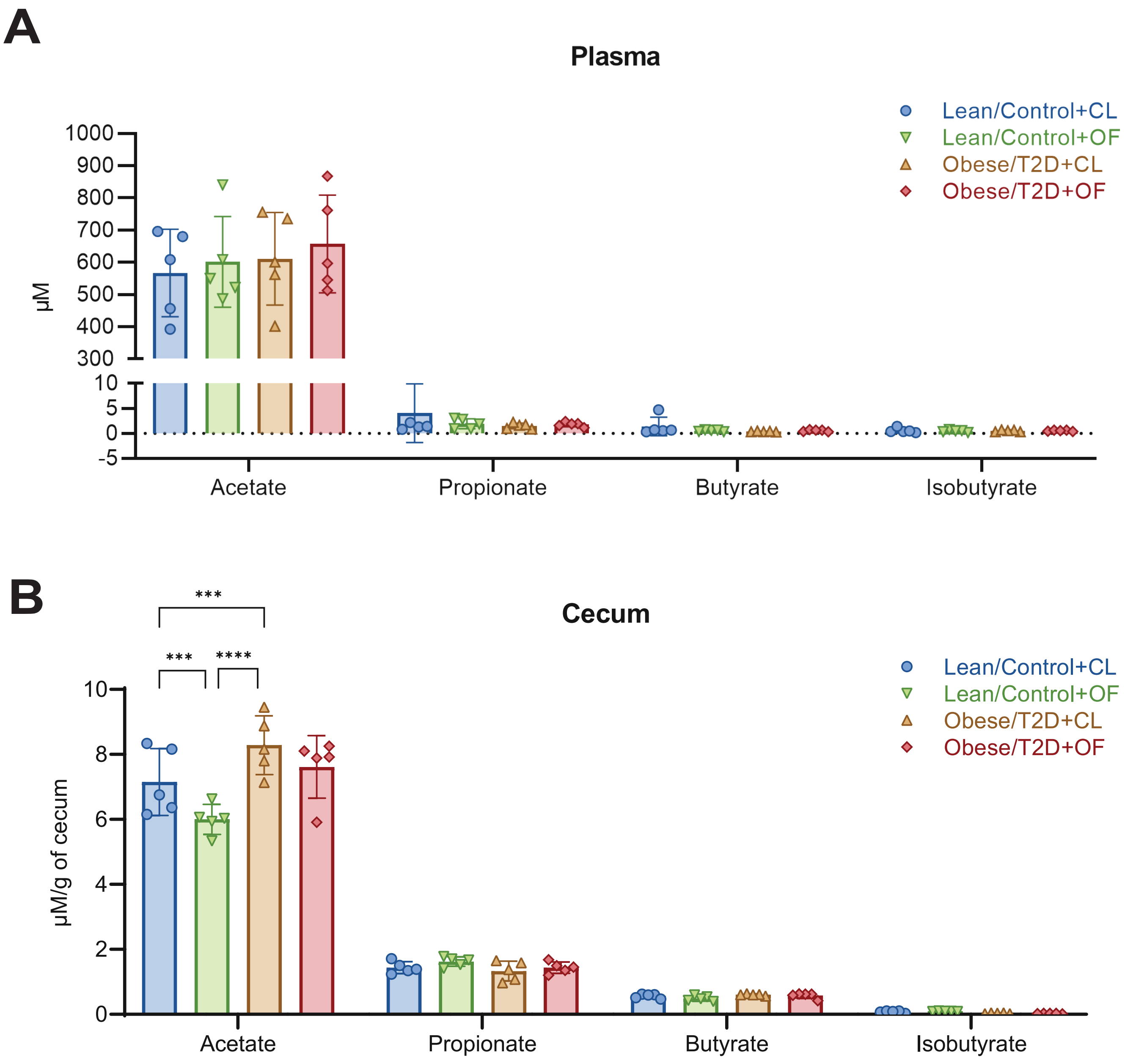

Figure S3. SCFA concentrations in plasma and cecum of obese/T2D mice given oligofructose. Short-chain fatty acids (SCFAs) were quantified by targeted metabolomics on samples isolated after two weeks of supplementation without infection as previously described. Bar graphs represent mean ± SD. n=5. Significance was identified using one-way ANOVA and Tukey's post-hoc multiple comparisons test. **P<0.01, ***P<0.001, ****P<0.001.

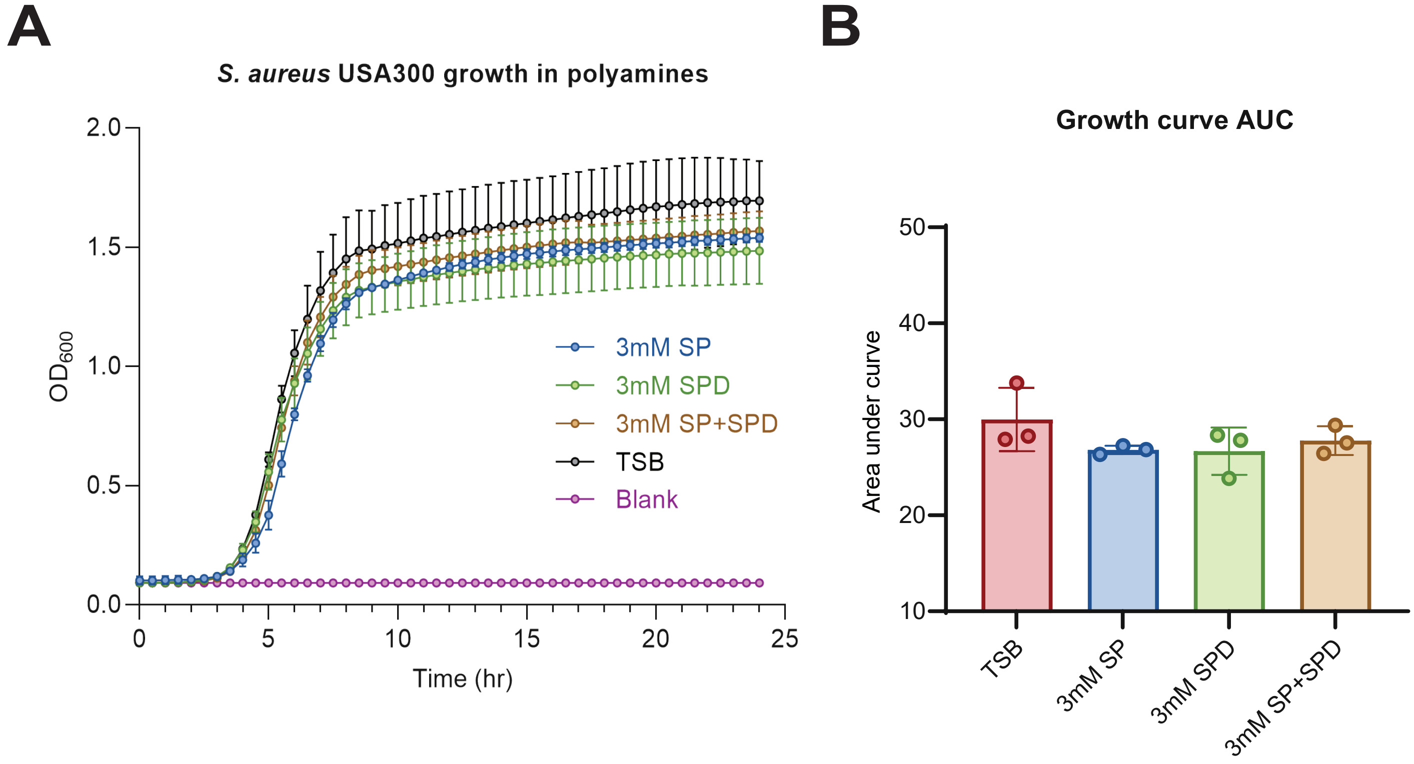

Figure S4. S. aureus growth in the presence of polyamines. A) S. aureus USA300 was incubated with various concentrations of spermine and spermidine in a 96-well plate and absorbance at 600 nm was monitored over 24 hours. SP (spermine), SPD (spermidine), TSB (tryptic soy broth) B) Area under curve was used to quantified overall growth across groups. Bar graphs represent mean ± SD. n=3. Significance was identified using one-way ANOVA and Tukey's post-hoc multiple comparisons test.
